# Looking for the needle in a downsized haystack: Whole‐exome sequencing unravels genomic signals of climatic adaptation in Douglas‐fir (*Pseudotsuga menziesii*)

**DOI:** 10.1002/ece3.7654

**Published:** 2021-05-17

**Authors:** Jan‐Peter George, Silvio Schueler, Michael Grabner, Sandra Karanitsch‐Ackerl, Konrad Mayer, Michael Stierschneider, Lambert Weissenbacher, Marcela van Loo

**Affiliations:** ^1^ Faculty of Science & Technology Tartu Observatory University of Tartu Tartu Estonia; ^2^ Department of Forest Growth, Silviculture and Genetics/Unit of provenance research and breeding Austrian Research Centre for Forests Vienna Austria; ^3^ Department of Forest Growth, Silviculture and Genetics Austrian Research Centre for Forests Vienna Austria; ^4^ Institute of Wood Science and Technology University of Natural Resources and Life Sciences (BOKU) Tulln Austria; ^5^ Center for Health & Bioresources AIT Austrian Institute of Technology GmbH Tulln Austria

**Keywords:** climatic adaptation, common garden experiment, Douglas‐fir, environmental association analysis, exome capture

## Abstract

1. Conifers often occur along steep gradients of diverse climates throughout their natural ranges, which is expected to result in spatially varying selection to local climate conditions. However, signals of climatic adaptation can often be confounded, because unraveled clines covary with signals caused by neutral evolutionary processes such as gene flow and genetic drift. Consequently, our understanding of how selection and gene flow have shaped phenotypic and genotypic differentiation in trees is still limited.
2. A 40‐year‐old common garden experiment comprising 16 Douglas‐fir (*Pseudotsuga menziesii*) provenances from a north‐to‐south gradient of approx. 1,000 km was analyzed, and genomic information was obtained from exome capture, which resulted in an initial genomic dataset of >90,000 single nucleotide polymorphisms. We used a restrictive and conservative filtering approach, which permitted us to include only SNPs and individuals in environmental association analysis (EAA) that were free of potentially confounding effects (LD, relatedness among trees, heterozygosity deficiency, and deviations from Hardy–Weinberg proportions). We used four conceptually different genome scan methods based on F_ST_ outlier detection and gene–environment association in order to disentangle truly adaptive SNPs from neutral SNPs.
3. We found that a relatively small proportion of the exome showed a truly adaptive signal (0.01%–0.17%) when population substructuring and multiple testing was accounted for. Nevertheless, the unraveled SNP candidates showed significant relationships with climate at provenance origins, which strongly suggests that they have featured adaptation in Douglas‐fir along a climatic gradient. Two SNPs were independently found by three of the employed algorithms, and one of them is in close proximity to an annotated gene involved in circadian clock control and photoperiodism as was similarly found in *Populus balsamifera*.

*Synthesis*. We conclude that despite neutral evolutionary processes, phenotypic and genomic signals of adaptation to climate are responsible for differentiation, which in particular explain disparity between the well‐known coastal and interior varieties of Douglas‐fir.

## Graphical abstract

16 provenances of Douglas‐fir were analyzed by using phenotypic and genomic data. Our data show strong signals of genomic adaptation to local climate conditions.

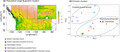

## 1 INTRODUCTION

Conifers have successfully occupied a large number of habitats and different climates during their past‐glacial histories, which allowed them not only to survive and establish in harsh environments but also to colonize ecoregions with optimal growing conditions (Farjon, 2010). As such, Douglas‐fir (*Pseudotsuga menziesii*) constitutes one of the most widespread conifers in western North America with a distribution from Southern California up to the Northern British Columbia (Gugger et al., 2010). Within its current range of occurrence, two distinct varieties are known, which appear genetically and phenotypically different: the coastal variety (also called *P. menziesii* var. *menziesii*) and the interior variety (*P. menziesii* var.*glauca)*. While the coastal variety is mainly found from central California to coastal British Columbia along the Pacific coast, the interior variety has its main south‐to‐north expansion from Wyoming/Montana over Alberta up to central British Columbia (Hermann & Lavender, 1990; Martinez, 1949). Both varieties hybridize when in contact, resulting, for example, in a 450‐km‐wide hybrid zone in British Columbia (Gugger et al., 2010) where also populations represented by both varieties and introgressed trees predominantly from the interior into the coastal variety were described (van Loo et al., 2015). The macrogeographic regions of both varieties differ substantially in climate with cool and moist climate conditions in the Pacific habitats toward more continental and dry growing conditions in the interior (Gugger et al., 2010). In addition, these habitats are also separated by the Great Basin and Columbia Plateau, respectively. Accordingly, several common garden experiments found intraspecific trait differences among Douglas‐fir provenances that were related to growth, physiology, and phenology and that showed associations with differences in seed source climate among provenance locations (Anekonda et al., 2004; Bansal et al., 2016; De La Torre et al., 2021; Kleiber et al., 2017; Malmqvist et al., 2017; Vangestel et al., 2018). While such trait–climate associations can indeed suggest patterns of local adaptation due to spatially varying selection (Leinonen et al., 2013), their interpretation can still be doubtful, because the climatic clines at which provenances occur often show parallelism with recolonization routes after population contraction and expansion (Nadeau et al., 2016). Consequently, the putative adaptive signal obtained from such trait analyses can be confounded by those that were left behind by neutral processes such as genetic drift and demographic processes, which have often formed genotypic clines as well (Caye et al., 2019; Günther & Coop, 2013).

Incorporating information from genetic markers has significantly improved our understanding of the role of neutral processes in population structuring of many plant species and has shed light into their postglacial migration histories (Hewitt, 1999). The main challenge, however, still remained, which is to distinguish truly adaptive regions in the genome from those that show strong divergence due to neutral genetic processes. Identifying true environmental associations and adaptive regions in the genome (e.g., from single nucleotide polymorphisms, SNPs) is of utmost importance, since only adaptive markers have the potential to assist tree breeding of more resistent genotypes and selection of climatically adapted genotypes for forest management under climate change (Grattapaglia et al., 2018; Harfouche et al., 2012; Neale & Kremer, 2011). Yet, one of the strongest limitations in disentangling adaptive from neutral genomic locations is acquiring a marker set, which is large and dense enough to cover the genome in a representative and unbiased fashion. This is particularly difficult in conifers such as Douglas‐fir with large genomes in the magnitude of several gigabases (De La Torre et al., 2014; Neale et al., 2017; Nystedt et al., 2013). Nevertheless, recent improvement in massive parallel sequencing and bioinformatics has made it possible to reduce the complexity of such genomes by reducing them to the protein coding region, which is called the *exome* (e.g., Neves et al., 2013). Exome capture in conifers results in massive reduction in genome complexity allowing to rapidly generate markers at high number and relatively low costs for a thorough study of genomic regions of interest (see, for instance, Capblancq et al., 2020; Suren et al., 2016; Thistlethwaite et al., 2017, for examples on conifers). Even though the exome represents only a small fraction of the total genome, these data are particularly suited for environmental association analyses (EAA), since adaptive candidate SNPs can be further examined when a gene model or annotated reference genome is available. Moreover, by using a subset of SNPs that show no significant deviation from neutrality expectation (as, e.g., examined with *F*_ST_ statistics), neutral population substructuring can be estimated as well.

In this study, we investigate a 40‐year‐old common garden experiment with 16 Douglas‐fir provenances from across its distribution by combining dendroclimatological methods with modern sequencing technique, which allowed us to generate a dense set of SNPs throughout the entire exome. Based on the first finding that provenances showed a strong association between growth traits (response of annual increment to summer temperature) and seed source climate, we used classical F_ST_ outlier approaches, Bayesian inference methods, and latent factor mixed modeling (LFMM) in order to identify SNPs with a truly adaptive signal for climate adaptation. We hypothesize that spatially varying selection has shaped the actual pattern of trait differentiation in Douglas‐fir despite a high amount of neutral covariance caused by genetic drift and gene flow.

## 2 MATERIALS AND METHODS

### 2.1 Common garden experiment

**FIGURE 1:**
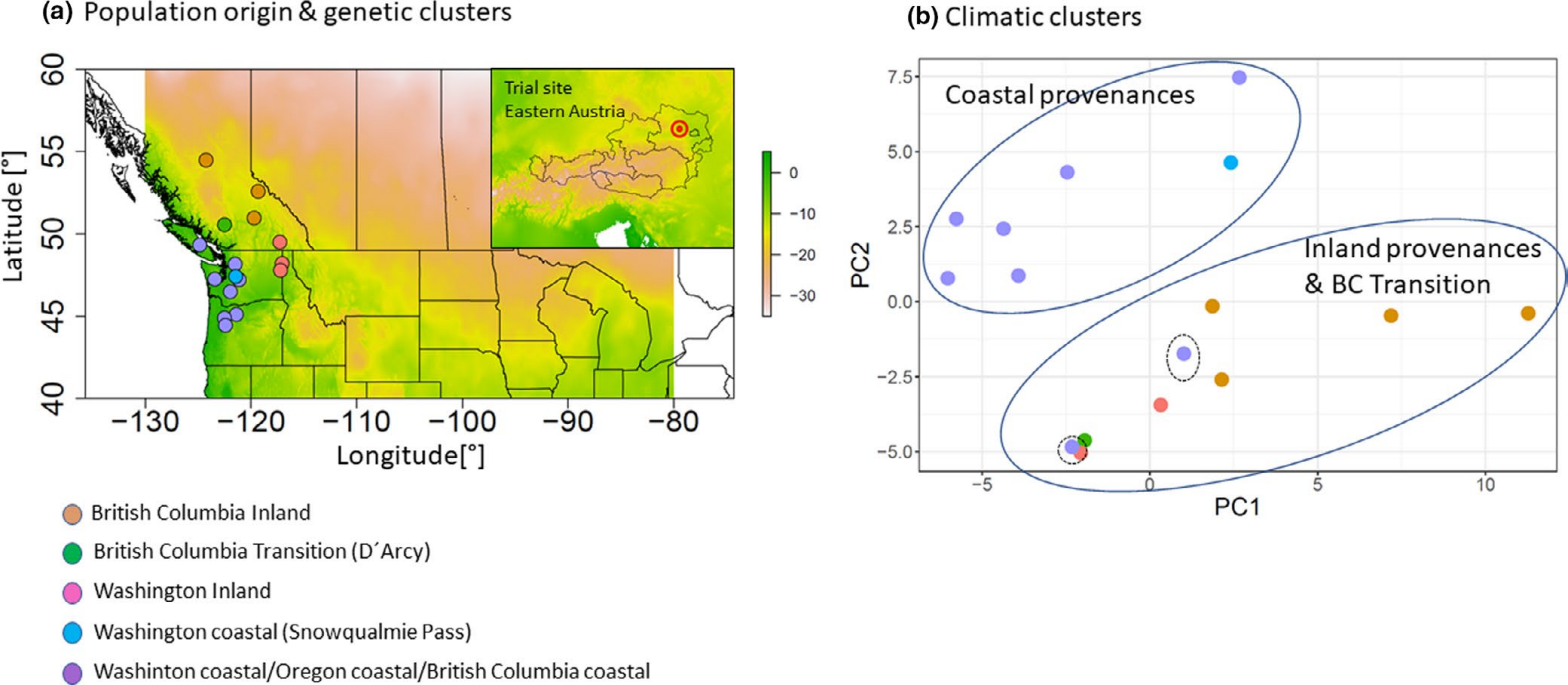
Geographic origin and genetic clusters of provenances (a), location of trial site (a small window), and climate clusters of provenances (b). Genetic clusters were colored by results of genomic PCA based on 1,500 randomly chosen markers

The analyzed trees originate from a block‐replicated common garden experiment established in 1976 in eastern Austria with a total of 49 native Douglas‐fir seed sources (provenances). The mean annual temperature of the trial site is 7.4°C, and mean annual precipitation is 650 mm (average over the period from 1961 to 1990; source: Austrian Central Station for Meteorology). Trees have been planted as two‐year‐old plants in a 2 × 2 m spacing, and each provenance was replicated three times in a random fashion within the trial. In 2012, two cores per tree were taken from a subset of 16 provenances originating from a wide part of the natural distribution encompassing Oregon, Washington, and British Columbia (see Figure 1, Table 1, and more information below). In brief, tree cores were taken at breast height for a total of 178 trees (9–15 per provenance and evenly sampled across the three blocks) and stored in plastic tubes until further processing in the laboratory. Cores were then cut into approx. 1.4‐mm‐wide cross sections with a double‐blade circular saw, placed on microfilms, and exposed to a 10 kV (24 mA) X‐ray source for 25 min. Thereafter, the obtained microfilms were analyzed using the software WinDENDRO 2009 (Regent Instrument, Quebec, Canada) by measuring annual increments to the nearest 0.001 mm for early‐ and latewood, respectively (see also George et al., 2019, 2017, 2015, for more information on the methodology). The 50% percentile of the density distribution between minimum and maximum density of each ring was used for the identification of earlywood–latewood boundaries (Fries & Ericsson, 2009; illustrated in Figure S1). The radial increment data from this common garden experiment, which is in detail described in George et al. (2019), were reanalyzed for this study, and therefore, additional needle samples for DNA analyses were taken from the same 178 trees in May 2019. Needles were shot from the lower part of the tree crowns by using a shot gun and stored in silica gel until DNA extraction.

**TABLE 1:** Overview of provenance origin and climate

Provenance ID	Name	Region	Latitude	Longitude	Altitude	MAT	MAP	*N*
AB	Alberni	British Columbia coastal	49.325	−124.85	150	9.2	2,161	10
NS	Nelson	British Columbia inland	49.5	−117.2667	750–900	6.5	941	13
FJ	Fort St. James	British Columbia inland	54.4833	−124.25	850	2	590	10
CM	Clemina	British Columbia inland	52.5833	−119.3167	900	3.9	854	9
AL	Adams Lake	British Columbia inland	50.9651	−119.7056	450–600	6.5	528	11
DA	D'Arcy	British Columbia transition	50.5567	−122.5	250	8.3	516	13
PG	Pine Grove	Oregon coastal	45.1	−121.3833	750	8.5	453	10
AQ	Abiqua Basin	Oregon coastal	44.8802	−122.506	600–750	8.3	2,191	11
CC	Cascadia	Oregon coastal	44.439	−122.41	600–750	10.2	1,947	10
ML	Matlock	Washington coastal	47.25	−123.4167	100	10.1	2,353	11
CE	Cle Elum	Washington coastal	47.2167	−121.1167	650	7	992	9
RD	Randle	Washington coastal	46.4847	−121.9491	300–450	9.3	1,604	13
DR	Darrington	Washington coastal	48.1601	−121.4968	900–1050	6	3,623	15
SP	Snoqualmie Pass	Washington coastal	47.4129	−121.4411	600–750	6.1	2,551	12
NP	Newport	Washington inland	48.2	−117.05	750	7.3	736	11
SK	Spokane	Washington inland	47.7833	−117.2	550–650	8.4	518	10
							Total	178

### 2.2 DNA analysis, probe design, and SNP calling

For each sample, genomic DNA was extracted from lyophilized needle tissue using a CTAB protocol (van der Beek et al., 1992) with minor modifications made for the processing of 96‐well plates. DNA concentration and quality were assessed with Qubit^®^ Quant‐iT dsDNA BR Assay kit and a Qubit 1 Fluorometer (Thermo Fisher). A total volume of 50 µl genomic DNA for each sample (average concentration: 24 ng/µl) was sent to the genotyping service provider RAPiD Genomics LLC (Gainesville, FL, USA) for sequencing.

Exome capture probes were designed as described in Neves et al. (2013): Briefly, DNA was sheared to a mean fragment length of 400bp, and fragments were end‐repaired, followed by incorporation of unique dual‐indexed Illumina adapters and PCR enrichment.

Intron–exon boundaries were designed by mapping transcripts from the published Douglas‐fir reference transcriptome from Howe et al. (2013) (https://www.ncbi.nlm.nih.gov/Traces/wgs/?val=GAEK01) to the Douglas‐fir reference genome assembly *Dougfir 1.0* (https://www.ncbi.nlm.nih.gov/assembly/GCA_001517045.1/#/st) from Neale et al. (2017). Only probes that hit once to the genome and that showed sufficient GC content were included. This resulted in 20 K probes that were found in 12,272 scaffolds with an average number of 1.6 probes per scaffold and a maximum number of 18 probes per scaffold. Sequence capture was performed using RAPiD Genomics proprietary chemistry and workflows. Samples were pooled equimolar and sequenced using a HiSeq 2x150.

After sequencing, *Trimmomatic* (Bolger et al., 2014) was used to remove sequencing adapters and the trimmed reads were mapped with BWA (Li & Durbin, 2009) using the default settings to the Douglas‐fir reference genome. SNPs within probes were identified with *FreeBayes* (Garrison & Marth, 2012, available at https://github.com/ekg/freebayes). *VCFtools* (Danecek et al., 2011) was used to store the identified SNPs for further downstream analysis in VCF format by applying the following filter: accepted mean depth across all individuals = 750, minimum total depth per individual = 3, percent of individuals allowed missing to retain a site = 0.4, minimum SNP quality = 10.

### 2.3 Consecutive SNP filtering and exclusion of trees with confounding effects

In order to create a genomic dataset that is free of sources, which could potentially affect the false‐positive discovery rate of adaptive SNPs, we used a restrictive and conservative filtering approach: As such, we removed all SNPs with a minor allele frequency < 0.05 and those SNPs that showed significant deviation from the Hardy–Weinberg expectation (threshold: 10^–6^). Furthermore, we performed pairwise linkage disequilibrium (LD) pruning between markers with a LD threshold of 0.2 in order to retain only unlinked SNPs for environmental association analysis. For this, we used the *snpgdsLDpruning* function implemented in the *SNPrelate* package in R (R Development Core Team, 2003).

Furthermore, kinship among individuals was estimated by calculating the identity‐by‐descent (IBD) methods of moments between all pairs of individuals using a subset of SNPs that was already corrected for LD (see section above). We retained only those trees that showed a kinship coefficient <0.25 to any other tree in the dataset. Trees that showed signals of heterozygote deficit (calculated as 1 − (Het_obs_/Het_Exp_) over all loci) were removed prior to analysis with a threshold of 0.1. Finally, we included only trees with an overall SNP call rate of 0.95 for subsequent analyses. All filtering steps were performed in R by using the packages *gdsfmt*, *SNPrelate* (Zheng et al., 2012), and *vcfR* (Knaus & Grünwald, 2017).

### 2.4 Climate data and trait analyses

#### 2.4.1 Climate data

Information on provenance climate was obtained from the ClimateNA database (Wang et al., 2016) under http://www.climatewna.com/. Latitude/longitude and elevation information for provenance origins was used to retrieve a total of 247 annual, seasonal, and monthly climate variables (reference period: 1961–1990). The full list of climate variables can be found in Appendix S1. In order to reduce the complexity of these data, the 247 variables were transformed into principal components and the first four PCs were standardized (i.e., expressed as standard deviations from the mean). These standardized climate PCs (hereafter called environmental PCs) were used for subsequent analyses of environmental association analysis (see Section 2.5 below).

#### 2.4.2 Phenotypic data

Growth traits for this dataset were derived from the study of George et al. (2019). Briefly, tree‐ring series were standardized using a 15‐year cubic smoothing spline in order to remove the biological age trend. The standardized series were aggregated to chronologies for each provenance by calculating the year‐to‐year biweight robust mean among trees in each provenance (Bunn, 2008). For the purposes of this study, we used the bootstrapped response functions that were applied to ring width chronologies of the same Douglas‐fir provenances in George et al. (2019). Response functions and in particular response coefficients between a growth trait (in our case, ring width) and trial site climate provide insights of the relative importance of an environmental variable for a trait (Lévesque et al., 2014; Zang & Biondi, 2013). However, when calculated at provenance level, bootstrapped response coefficients can be related to provenance source climate in order to unravel genecological clines (i.e., some provenances may react more sensitive to an environmental factor than others as a result of local adaptation). In this study, we used the response of earlywood increment to July temperature at trial site as trait of interest because of two reasons: First, the data in George et al. (2019) unraveled significant differentiation among Douglas‐fir provenances for this trait; and second, growth response in earlywood has been shown to be significantly involved in drought response and subsequent survival in Douglas‐fir (Martinez‐Meier et al., 2008), which suggests that this trait most likely harbors signals of spatially varying selection among provenances. Multiyear correlations between standardized earlywood time series and July temperature at trial site between 1979 and 2011 were bootstrapped by using the R packages *bootRes* (Zang & Biondi, 2013) and *dplR* (Bunn, 2008).

### 2.5 Outlier detection and environmental association analysis (EAA)

To distinguish SNP markers throughout the exome that have a putative adaptive signal for an environmental factor from nonadaptive (neutral) SNPs, we used four different algorithms. These are based either on F_ST_ outlier detection methods (Arlequin & BayScEnv) or on correlations between allele frequencies and environmental variables (BayEnv2 and LFMM2). Regardless of the algorithm that is applied, controlling for variation in allele frequencies that is simply caused by neutral evolutionary processes such as genetic drift or gene flow is a crucial step for identifying adaptive candidate SNPs (Rellstab et al., 2015). Since the fraction of adaptive SNPs is usually small compared with all analyzed SNPs, adjusting the results carefully for false‐positive discovery rates is of utmost importance. For that reason, we selected four population genomic programs (Arlequin, BayScEnv, BayEnv2, and LFMM2), which have implemented various testing strategies in order to control for confounding effects and false‐positive errors.

The coalescent approach implemented in *Arlequin* 3.5 (Excoffier & Lischer, 2010) compares locus‐specific patterns of population differentiation with a null distribution, which is simulated under the assumption of a hierarchical island model (Excoffier et al., 2009). *p*‐Values are estimated locus‐wise from the joint distribution of heterozygosity and *F*_ST_. If the locus‐specific F_ST_ exceeds the confidence boundaries of the global observed *F*_ST_, the respective locus is considered to be an outlier and likely to be under selection. Since we observed significant population substructure (see Section 3) in our dataset, we chose the hierarchical island model instead of the finite island model in order to reduce the number of false‐positive candidate SNPs, since the confidence intervals around a given F_ST_ value become narrower, the more the populations are sampled (see Excoffier et al., 2009). Groups for the hierarchical island models were defined based on the first two principal components obtained from 1,500 randomly chosen neutral SNPs with the snpgdsPCA function in the *SNPRelate* package in R. We used 200,000 simulations in Arlequin vers. 3.5 with 10 simulated groups and 100 demes in each group. SNPs were considered to be under selection when their observed *F*_ST_ exceeded the 5% upper quantile of the simulated null distribution.

BayScEnv (Villemereuil & Gaggiotti, 2015) is a *F*_ST_‐based genome scan method that incorporates also environmental differentiation. The algorithm tests a model of local adaptation with environmental differentiation (i.e., the parameter **g** in Villemereuil & Gaggiotti, 2015) against two alternative models: (a) the neutral model consisting largely of demographic effects and (b) the locus‐specific model that includes locus‐specific effects other than selection due to environmental differentiation. Posterior error probabilities for choosing the model of local adaptation in favor of the two alternative models are calculated by means of MCMC simulations, and results are also adjusted for false discovery rates by providing *q*‐values for each locus. We used 20 pilot runs each with a length of 2000 and a burn‐in of 50,000 with a thinning interval of 10. Furthermore, a prior probability of 0.1 was used for the parameter *pi* (assuming every 10th locus harbors a non‐neutral signal), while a prior probability of 0.5 was used for the parameter *p* (assuming around 50% of all loci are associated with an environmental variable). Acceptance rates and convergence of the MCMC chain were inspected after each model run and for each of the four environmental PCs by using the *coda* package in R.

In contrast to the F_ST_‐based outlier detection method, *Bayenv2*.*0* (Günther & Coop, 2013) calculates standardized allele frequencies and tests for correlations between environmental variables and those frequencies. Neutral and spurious correlations arising from shared population history and gene flow are controlled for by including a covariance matrix obtained from a subset of unlinked and putatively neutral SNPs. We used a subset of 1,500 randomly chosen unlinked SNPs for matrix estimation and ran Bayenv2.0 with 100,000 iterations. Subsequently, all identified SNPs for each of the four environmental PCs with a Bayes factor >30 (Jeffreys, 1961) were considered as candidates for environmental association analysis. Since the algorithm in Bayenv2.0 is based on MCMC simulations, each run was repeated three times in order to ensure that results remain robust. We additionally calculated spearman rank correlation coefficients in addition to Bayes factors for each SNP in order to ensure that detected candidates were correctly identified and not confounded by outliers (see Table 2 and recommendation in Günther & Coop, 2013).

**TABLE 2:** Utilized algorithms and chosen thresholds for SNP identification

Algorithm	Method for accounting for neutral processes	Climate data included in calculations	Threshold	Total number of outlier SNPs	Climate PC1	Climate PC2
Arlequin	*F*_ST_ hierarchical island model	No	−log_10_ (1−*F*_ST_ quantile) > 1.3	1,148 (29)		
BayScEnv	*F*_ST_ null model	Yes	−log_10_ (*Q*‐value) > 1.3	4	4	0
BayEnv2	Matrix estimation from neutral SNPs	Yes	BF > 30; |rho| > 0.4	28	10	18
LFMM2	*K* latent factors from PCA	Yes	−log_10_ (*p*‐value) > 1.3	1,921 (4)	1,082 (2)	908 (2)

Finally, we used a regression model combining fixed (i.e., environmental) and latent model effects (i.e., neutral population structure). The model has the form:

(1) $$Y=XB^{T}+W+E$$

with *Y* being the response genotype matrix, *XB^T^* the matrix of fixed effects (climate PCs), *E* the matrix of residual errors, and *W* the latent matrix (see Caye et al., 2019). *W* determines the confounding effect of neutral population structure and is determined by *k* latent factors, where *k* defines the number of ancestral populations as obtained from a subset of putative neutral loci. As an approximation of *k*, we used the scree plot obtained from principal component analysis performed on the 1,500 randomly chosen SNPs described above. Calculations were carried out using the *lfmm2* function in the LEA package in R (Frichot & François, 2015).

### 2.6 Adaptive signal of outlier SNPs and functional interrogation

In order to further interrogate the identified candidate SNPs, we first defined a set of consensus variants. Consensus SNPs were defined as those SNPs that were independently detected by at least two of the four chosen methods according to thresholds in Table 2. Subsequently, we functionally interrogated these SNPs with the help of the annotated Douglas‐fir reference genome (Psme.1_0) available under https://treegenesdb.org/FTP/Genomes/Psme/v1.0/. We used the integrative genomics viewer (Robinson et al., 2011) and explored scaffold‐wise whether consensus SNPs were located within known genes or situated nearby to genes. Finally and in order to prove whether the unraveled consensus SNPs have the ability to discriminate between provenances adapted to different climates, the first and second eigenvectors for each tree were calculated based on consensus SNPs and, for comparison, based on an equal number of randomly chosen neutral SNPs, respectively. For this, the *snpgdsPCA* function from the SNPRelate package in R was employed.

### 2.7 Isolation by climate versus isolation by distance and isolation by colonization

In order to disentangle the various sources that could have caused genomic and phenotypic differentiation among Douglas‐fir populations, we performed redundancy analysis (RDA). RDA uses a multiple linear regression method in order to model allele frequencies as a function of independent explanatory matrices. As independent matrices, we included five different predictors: the first two climate PCs, geographic information (Lat, long), and information on shared colonization history. For the latter, we used the STRUCTURE algorithm (Pritchard et al., 2000) and estimated ancestry proportions (*Q*‐values) for each tree testing different scenarios of putative ancestral populations (*K*: 1–8) for the neutral subset of 1,500 SNPs, which was described above. The most likely number of ancestral populations was chosen based on the minimum cross‐entropy criterion (Alexander & Lange, 2011). For this, we visually inspected the cross‐entropy plot and determined the number of populations where the cross‐entropy reached a plateau. This revealed a most likely *K*‐value of two, and populations were divided into a western and eastern cluster (see Section 3 and Figure S2). Latitude, longitude, and *Q*‐values were z‐transformed prior to analyses as was done for the climate PCs. We performed different sets of RDAs on the complete SNP dataset (17,489 SNPs) and subsequently for the consensus outlier SNP set described in Section 2.6: Combined models included either both climate PCs, geographic information (latitude and longitude), or *Q*‐values, respectively. In individual models, we also tested for the effect of every single predictor separately (i.e., either climate PC1, climate PC2, latitude, longitude, or *Q*‐value). For this analysis, the *vegan* package in R was employed (Oksanen et al., 2020).

## 3 RESULTS

### 3.1 SNP dataset

Within the 20K probes that were designed for exome capture, a total of 90,979 SNPs were successfully called with *Freebayes* and passed the applied quality filter as described in Section 2.2. Since a large proportion of these 90,979 SNPs had either a minor allele frequency <0.05 or a call rate <0.95, 41,009 SNPs were consequently removed and not considered for downstream analysis. 4,269 markers showed significant deviations from the Hardy–Weinberg proportion and were excluded from analyses accordingly. Finally, testing for linkage disequilibrium among markers revealed 17,489 SNPs, which were physically unlinked, and only these SNPs were included in all subsequent analysis steps.

From the 178 trees, 5 showed significant heterozygosity deficiency probably as a result of inbreeding and were hence excluded from subsequent analyses. There was no significant family structure among trees within provenances as revealed by IBD methods of moments so that all remaining 173 trees were included in environmental association analysis.

### 3.2 Climatic groups, population structure, and growth response functions

The first two principal components of the 250 long‐term climatic variables together explained 79.9% of variation (Figure 1). The first principal component (hereafter called climate PC1) was strongly related to temperature variables (correlation with mean annual temp. −0.99 and with minimum temperature of coldest month: −0.97) and length of growing season (correlation with number of growing degree days > 5°C = 0.88). The second component (climate PC2) was strongly correlated with precipitation regime at seed origin (*r*_MAP _= 0.94; *r*_summer:heat mositure index _= 0.81). The third and fourth PCs explained less variation and were related to solar radiation and annual snow precipitation, respectively (Appendix S2). As expected, provenances could be roughly divided into two main groups when plotted against the first two climate PCs, that is inland and coastal provenances (Figure 1b). Based on the subset of 1,500 randomly chosen SNPs, five population clusters were unraveled: (a) provenances from northern inland British Columbia (Fort St. James, Clemina, Adams Lake), (b) the three inland provenances from interior Washington and Southern British Columbia (Newport, Spokane (both WA) and Nelson (BC)), (c) coastal provenances from British Columbia, Washington, and Oregon (Alberni, Matlock, Cle Elum, Pine Grove, Randle, Darrington, Abiqua Basin, and Cascadia), (d) a single provenance cluster Snowqualmie Pass (WA), and (e) another single provenance cluster including provenance D'Arcy in the transition zone between coastal and inland British Columbia (Figure 1a).

**FIGURE 2:**
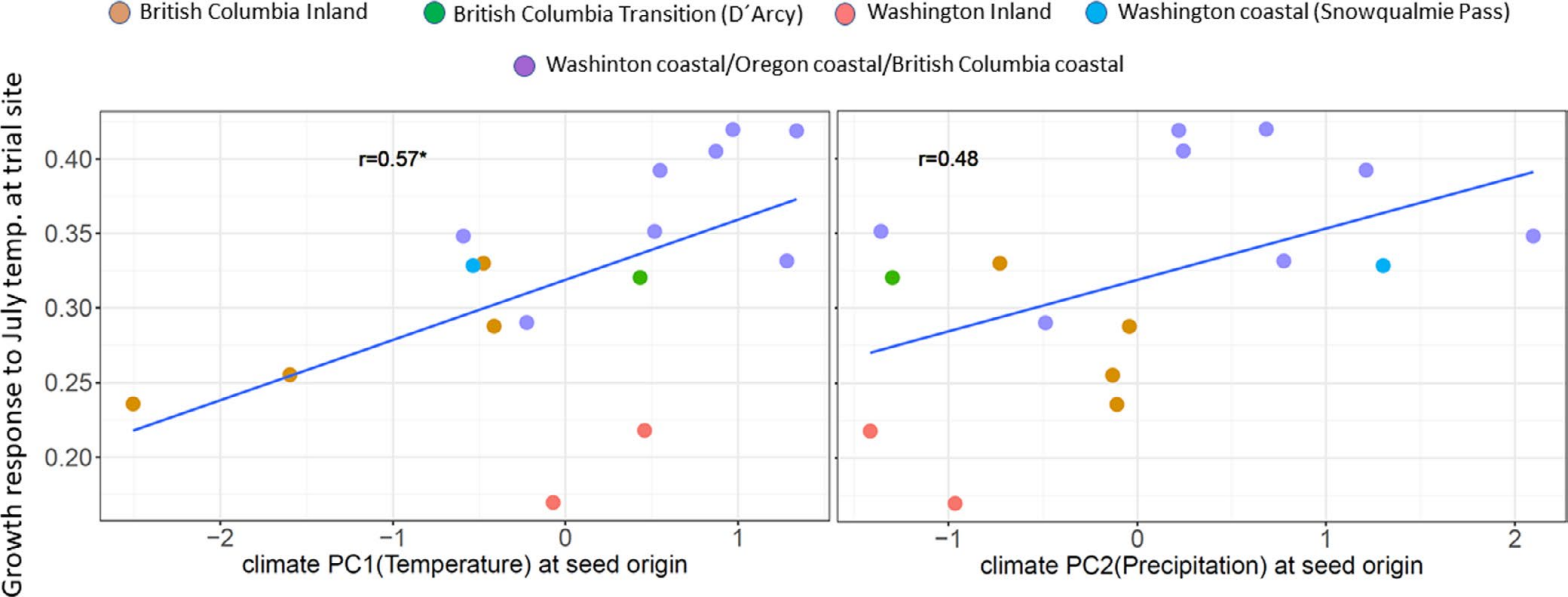
Phenotypic differentiation among provenances and relationship between response of earlywood to summer temperature in the common garden (*y*‐axis) and climate at seed origin (*x*‐axis)

Response of earlywood growth to July temperature at the trial site varied significantly among provenances. In general, inland provenances from British Columbia and Washington had lower response coefficients than their coastal counterparts (Figure 2). Response coefficients varied between 0.17 (Newport, WA) and 0.42 (Cascadia, OR). Differences among provenances were statistically significant as suggested by 95% confidence intervals of the bootstrapped coefficients. When regressed against climate PC1 and PC2, the relationship was significant with a stronger positive earlywood response toward warmer provenance origins (*r* = .57; Figure 2a) and toward wetter provenance origins (*r* = .48; Figure 2b). There were no significant relationships to climate PC3 and climate PC4, and consequently, only the first two climate PCs were considered for environmental association analysis.

### 3.3 Outlier SNPs and environmental associations

**FIGURE 3:**
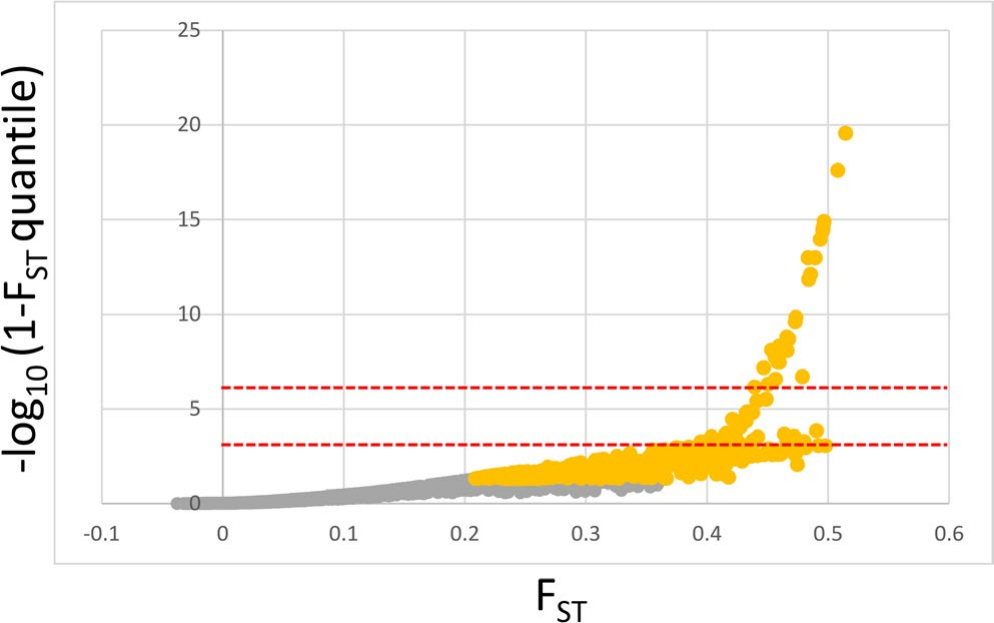
*F*_ST_ outliers according to the hierarchical island model implemented in Arlequin 3.5. Yellow points show SNPs outside the upper 5% quantile of *F*_ST_ distribution. Red dashed lines show *p* < .001 and *p* < 5.54e−06 thresholds, respectively. The latter represents the 5% significance threshold after Bonferroni adjustments for multiple testing

**FIGURE 4:**
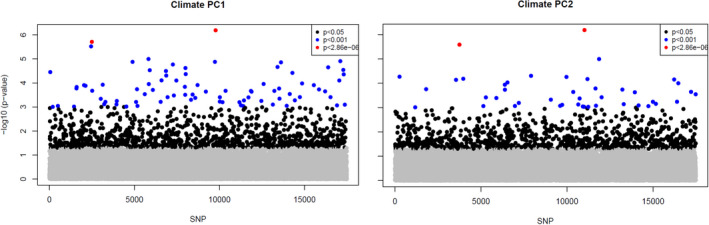
Manhattan plots for marker p‐values as obtained from LFMM2. Black dots show markers with *p* < .05, blue dots with *p* < .001 and red dots show all SNPs that were still significant after adjusting for multiple testing

**FIGURE 5:**
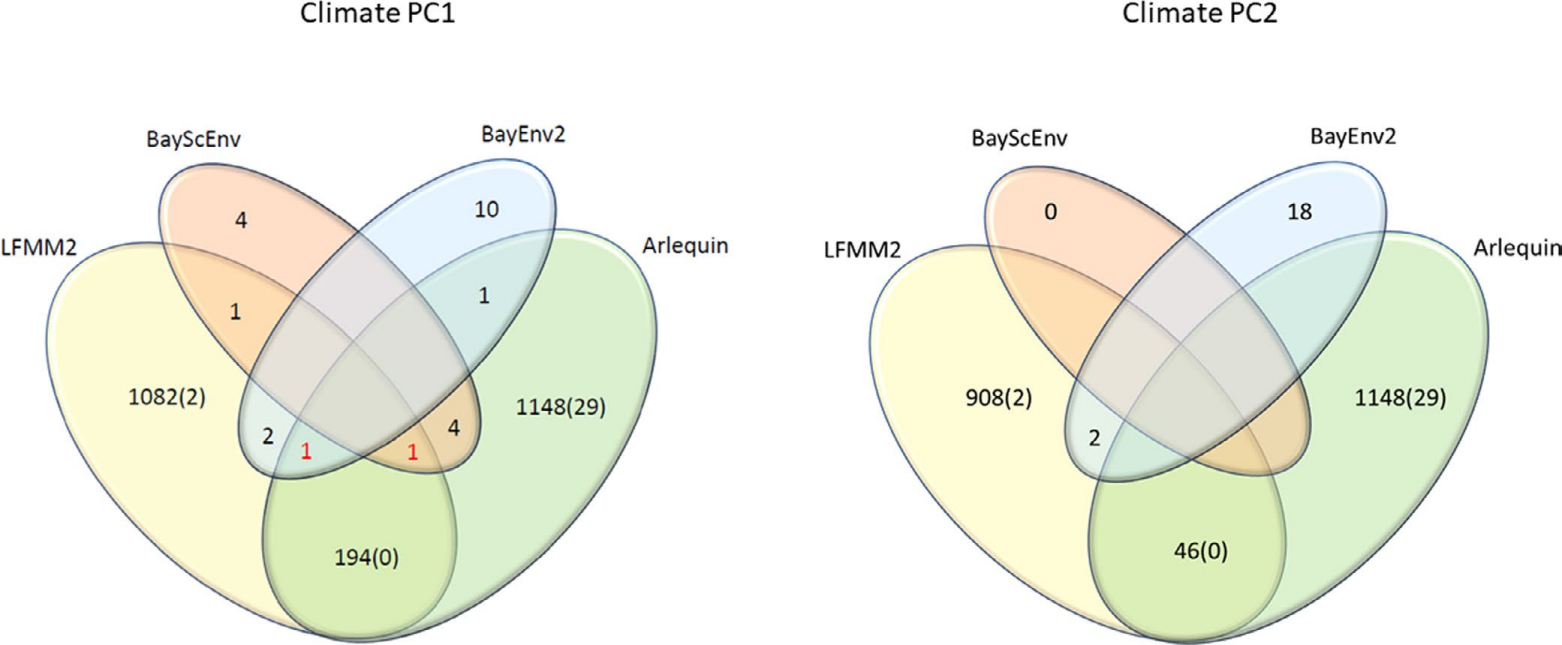
Venn diagrams for identified SNPs among the different algorithms. Numbers in red show SNPs that were discovered three times. Numbers in brackets indicate SNPs still significant after adjusting for multiple testing

The total number of outlier SNPs and those associated with climate varied significantly among detection methods. Arlequin and LFMM2 found a total of 1,148 and 1,082 SNPs, which showed signals of selection and association with climate PC1, respectively. However, when corrected for multiple comparisons, only 29 and 2 SNPS, respectively, passed the adjusted *p*‐value threshold (−log_10_ corrected = 5.54). BayEnv2 revealed a total of 10 SNPs associated with climate PC1 and BayScEnv exhibited 4 SNPs (Figures 3 and 4a, Table 2). 194 SNPs were commonly found by Arlequin and LFMM2, 2 SNPs were found by LFMM2 and BayEnv2, each 1 between Arlequin and BayEnv2 and LFMM2 and BayScEnv, and all four SNPs detected with BayScEnv were also listed as outliers in Arlequin. As the most promising candidates, SNPs #15099 and #78509 appeared independently as outliers associated with temperature in three of the four employed programs (Figure 5a).

For climate PC2, LFMM2 detected 908 SNPs (2 SNPs still significant after Bonferroni correction), BayEnv2 detected 18 SNPS, while no significant SNPs were detected by BayScEnv. 46 SNPs that appeared as outliers in Arlequin were also shortlisted in LFMM2, and SNPs #51115 and #69292 were commonly detected by LFMM2 and BayEnv2. However, for climate PC2 no SNP appeared to be shortlisted more than twice.

### 3.4 Interrogation of putative adaptive SNPs

A total of 18 consensus SNPs (11 for climate PC1 and 7 for climate PC2) were selected for functional interrogation and spatial frequency analysis. All 18 SNPs were at least found independently by two or three of the used algorithms when the thresholds described in Table 2 were applied. However, since a large number of common SNPs were found between LFMM2 and Arlequin (194 and 46, respectively), we selected only the top five candidates ranked by the ‐log_10_ LFMM p‐value for both climate PCs, respectively.

Out of the 18 consensus SNPs, six were located directly within annotated genes, five were situated in close proximity to known genes (less than 1,000 bp up‐ or downstream), and the remaining seven were located more distantly from annotated genes (>9 kb). Ten genes had known functions, and these encompassed mostly well‐known proteins, enzymes, and transcription factors involved in signaling, DNA‐binding, and methylation (Table 3). Most interestingly, for SNP #78509 which was independently found by three of the four algorithms as outlier, a circadian clock protein involved in photoperiod sensing in *Arabidopsis thaliana* was found to be coded by Douglas‐fir gene *PSME_16548*.

**TABLE 3:** Functional annotation and genomic position of outlier SNPs

SNP‐ID	*F*_ST_	Environmental factor (Climate PC)	Ref	Alt	Position	Psme.1_0 ID	Homolog	Functional annotation
13432	0.318517	Temperature	G	T	Within	PSME_50311	XP_010254239.1	Protein TIFY 8‐like
30680	0.285955	Temperature	C	A	Within	PSME_15966	XP_021822298.1	Cysteine synthase, chloroplastic/chromoplastic‐like
70743	0.359548	Temperature	A	G	Within	PSME_12112	XP_006848390.2	hemK methyltransferase family member 2
69642	0.303341	Temperature	T	G	Within	PSME_27253	AbisacEGm005830t1	Unknown
58318	0.339572	Precipitation	A	G	Within	PSME_28492	XP_021641448.1	Transcription factor FAMA‐like
31046	0.39123	Precipitation	T	C	Within	PSME_50228	MA_9245337g0020	Unknown
89806	0.30836	Temperature	A	T	<1,000 bp	PSME_21916	XP_022768877.1	40S ribosomal protein S2–2‐like
12985	0.364605	Temperature	T	C	<1,000 bp	PSME_36263	XP_020271476.1	60S ribosomal protein L31
78509	0.412301	Temperature	T	C	<1,000 bp	PSME_16548	XP_006844718.1	WD repeat‐containing LWD1
60539	0.379081	Precipitation	G	C	<1,000 bp	PSME_08707	GACG01002090.1	Unknown
51115	0.205466	Precipitation	C	A	<1,000 bp	PSME_16051	XP_023549543.1	Bifunctional UDP‐glucose 4‐epimerase and UDP‐xylose 4‐epimerase 1
15099	0.297343	Temperature	T	C	>9,000 bp	PSME_21319	AbisacEGm027507t1	Unknown
20020	0.311698	Temperature	G	T	>9,000 bp	PSME_13222	AbisacEGm021661t1	Unknown
71701	0.388778	Temperature	C	T	>9,000 bp	PSME_33882	XP_022769023.1	Inositol‐pentakisphosphate 2‐kinase‐like
69292	0.281179	Precipitation	G	C	>9,000 bp	PSME_27253	AbisacEGm005830t1	Unknown
57319	0.265295	Precipitation	G	A	>9,000 bp	PSME_43532	XP_006844754.1	Chaperone protein ClpD, chloroplastic
45651	0.176026	Temperature	A	T	>9,000 bp	NA		
17435	0.261599	Precipitation	T	A	>9,000 bp	NA		

Note

Functional annotation refers to functions obtained from NCBI gene database (https://www.ncbi.nlm.nih.gov/gene/). “NA” indicates that no annotated gene was located on the respective scaffold.

**FIGURE 6:**
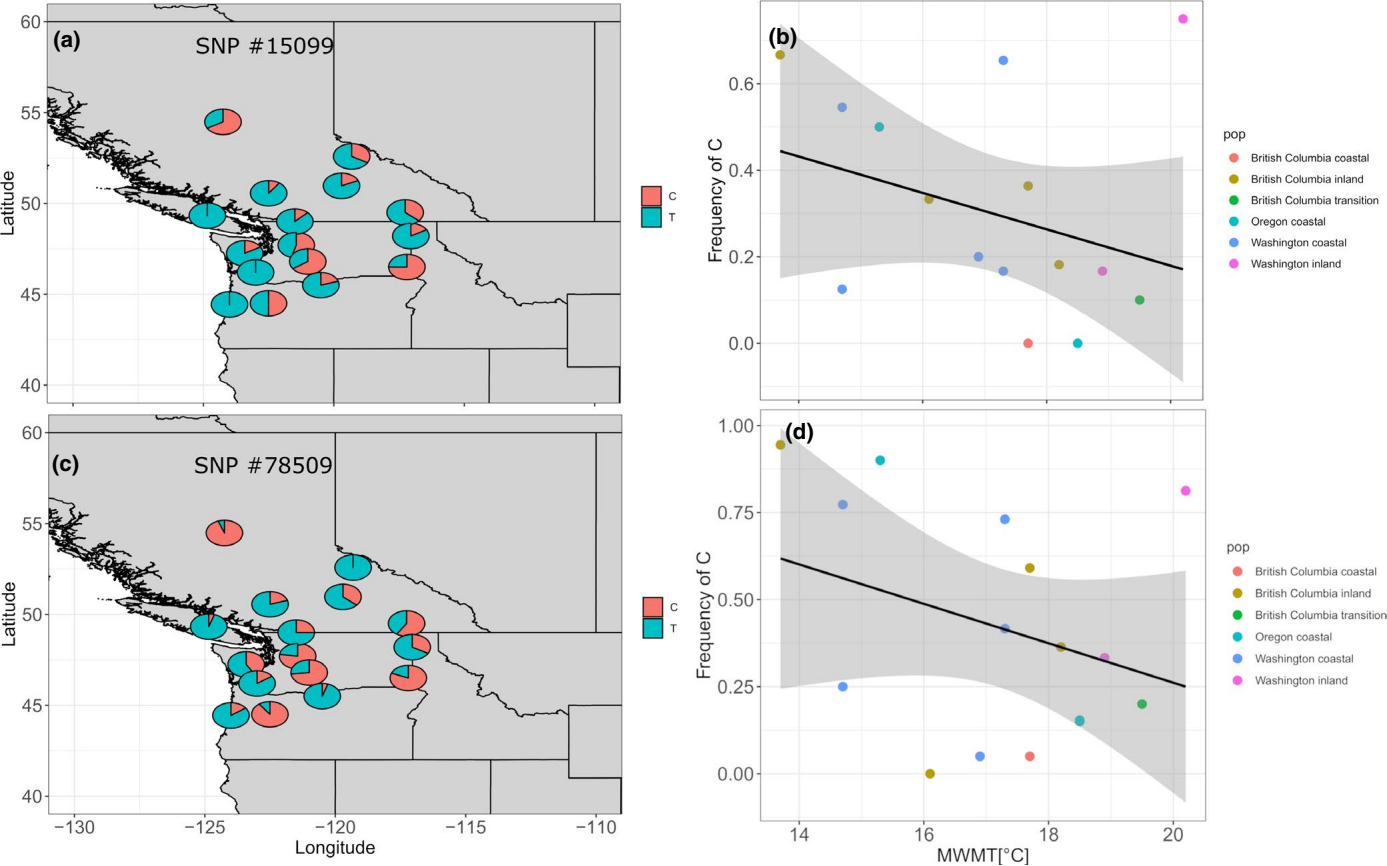
Allele frequencies of the two triple‐found SNPs #15099 (a–b) and #78509 (c–d). Graphs in (b) and (d) show simple regression of the alternative allele frequency against mean warmest month temperature at trial site

Finally, our two candidate SNPs #15099 and #78509, which were three times independently identified as truly adaptive outliers, showed at least by trend differentiation in space, since the alternative allele increased in frequency for both SNPs toward the inland and those toward colder provenance locations (Pearson's correlation coefficients between allele frequencies and mean warmest month temperature were −0.31 and −0.32, respectively; Figure 6).

**FIGURE 7:**
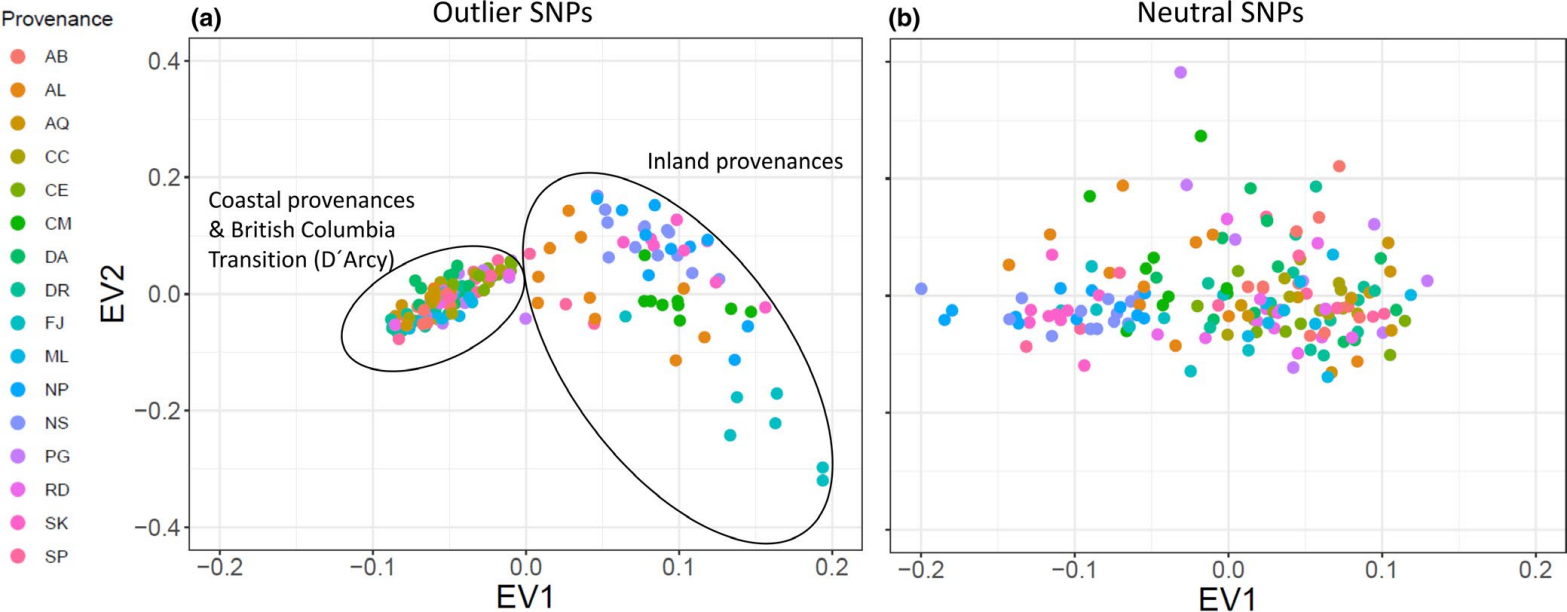
Clustering of populations according to (a) the 18 outlier SNPs shown in Table 3 and (b) 18 randomly chosen neutral SNPs. EV1 and EV2 are eigenvectors obtained from snpgdsPCA function

When comparing the first two eigenvectors based on the 18 consensus SNPs with eigenvectors obtained from an equal number of randomly chosen neutral SNPs, provenances could be clearly separated into inland and coastal provenances, whereas no clear differentiation was observed for 18 randomly chosen SNPs (Figure 7).

### 3.5 Isolation by climate versus isolation by distance and isolation by colonization

Redundancy analysis revealed no significant effect of the tested predictors for among‐population differentiation regardless of whether the total set of SNPs was used or the 18 adaptive consensus SNPs (Table 4). The proportion of explained variance ranged between 0.007 (east–west ancestry) and 0.013 (climate) in the combined models and between 0.002 (longitude) and 0.01 (climate PC1) in the individual models. Only climate PC1 (temperature) showed a moderate significant effect for among‐population differentiation when tested on the outlier SNP set (significant at *α* < 0.1).

**TABLE 4:** Summary statistics from redundancy analysis

	All SNPs		18 consensus SNPs	
	*R* ^2^	*p*‐Value	*R* ^2^	*p*‐Value
Combined model				
Climate (PC1 + PC2)	.01	.26	.01	.28
Geography (Lat + Lon)	.01	.90	.01	.73
Ancestry (*Q*‐values for *K* = 2)	.01	.25	.01	.26
Individual model				
Climate PC1	.01	.10	.01	.091*
Climate PC2	.01	.91	.00	.83
Latitude	.01	.42	.01	.39
Longitude	.00	.98	.00	.91
Ancestry	.01	.93	.00	.79

* Significant at *α* < 0.1.

## 4 DISCUSSION

In this study, we combined observations from a common garden experiment with genomic information in order to unravel polymorphisms, which could have caused the strong phenotypic cline provenances showed after 40 years of growing. While such old common garden experiments are rare for most tree species, their retrospective analysis using dendroclimatology among other approaches can shed light in evolutionary history and adaptation patterns, because a high number of repeated observations are naturally archived in tree cores. Although this study is not the first attempt that linked molecular genomics with tree‐ring data (Heer et al., 2018; Housset et al., 2018; Trujillo‐Moya et al., 2018), it is one of the very first environmental association analyses incorporating phenotypic data for the economically important Douglas‐fir, which is recently attracting attention outside its natural range due to its ability to cope with climate warming and drought (Eilmann & Rigling, 2012). Hence, we will discuss our findings also in light of adaptive forest management.

### 4.1 Growth response of trees after four decades in the common garden

The sixteen analyzed provenances showed a strong phenotypic cline with temperature and precipitation at seed origin. Provenances with earlywood growth strongly responding to July temperature at trial site originated mainly from the warmer and wetter coastal sites in British Columbia, Washington, and Oregon, whereas less responsive provenances came from colder and drier inland sites. Many other studies have revealed such a differentiation pattern between the coastal variety and interior variety in terms of either productivity (Eilmann et al., 2013), physiology (Anekonda et al., 2004), or even stress response (Kleiber et al., 2017). Our findings could indeed add evidence that coastal varieties are generally more productive, since earlywood response to summer temperature is a strong indicator for overall productivity. Earlywood cells in conifers mainly ensure that water demand in the crown will be sufficiently covered during phases of high evapotranspiration and trees with larger earlywood fractions can consequently allocate more carbon (Björklund et al., 2017). The climate at our trial site already represents the dry margin of the natural distribution of Douglas‐fir and is prone to extreme summer droughts (George et al., 2019). Hence, we can assume that trees have been growing under stressful conditions in most of the years. The study by George et al. (2019) in which the same trees have been analyzed also demonstrated that provenances from warmer locations had less growth reductions during years with extreme water deficit, whereas colder inland provenances had the highest reductions in annual increment. However, it has yet to be confirmed whether the higher drought tolerance of coastal provenances (expressed as ratio between growth during the drought year compared with a predrought period) really mirrors the ability of trees to withstand drought or simply mirrors adaptive growth patterns, which could come at the cost of lower recovery or higher mortality after drought periods (Montwé et al., 2015).

### 4.2 Outlier SNPs and the role of selection across a steep environmental cline

One of the main goals of this study was to identify polymorphisms, which show true signals of adaptation to climate and which are not confounded by neutral processes such as gene flow, drift, and migration. The analyzed provenances showed strong genetic substructuring which partly coincided with phenotypic differentiation (Figures 1 and 2). This makes disentangling adaptive from nonadaptive signals particularly challenging (Ahrens et al., 2018; Nadeau et al., 2016; Rellstab et al., 2015). To solve this problem, we employed four conceptually different algorithms, which implement various methodologies for taking neutral processes into account. Each of them identified a certain numbers of outliers or associations, respectively. The genome scan method in Arlequin identified the highest number of SNPs, which is not surprising, since Arlequin does neither specifically associate polymorphisms with climatic factors nor does it take environmental differentiation into account. Hence, the identified SNPs are probably loaded with adaptive signals from other environmental drivers than temperature and precipitation. Surprisingly, after correcting for multiple testing only a very small number of SNPs were still standing out as truly adaptive and represented only between 0.01% (LFMM) and 0.17% (Arlequin) of the analyzed SNPs. Although direct comparison with other studies is complicated due to varying experimental settings and sample size (see, e.g., Ahrens et al., 2018), adaptive SNPs in other genera such as *Fagus* (Pluess et al., 2016), *Populus* (Fahrenkrog et al., 2017), *Alnus* (De Kort et al., 2014), and *Pinus* (Ruiz Daniels et al., 2019) comprised between 1.6% (Fahrenkrog et al., 2017) and 11% (Pluess et al., 2016) of all SNPs that were analyzed. Moreover, when the data were further aggregated to consensus SNPs, which were found by more than one algorithm, only 11 candidates out of 17,489 (0.06%) were detected for temperature and 7 (0.04%) for precipitation regimes. Since the analyzed provenances exhibit a strong pattern of population substructuring into clearly delimited clusters, we strongly presume that this neutral variation is the most important cause for this finding. In particular, provenances D'Arcy (BC) and Snowqualmie Pass (WA) represented strongly isolated subpopulations based on their neutral genetic pattern (Figure  1a), although their phenotypic signal fit well into the observed environmental cline for both climate PCs (Figure 2). Provenances Pine Grove (Oregon coastal) and Cle Elum (Washington coastal) are climatically more similar to inland provenances (cold and comparatively dry), but share demographic history clearly with the other coastal varieties (Table 1, Figure 1). Phenotypically, both provenances had indeed lower response coefficients compared with the remaining coastal provenances, which demonstrates that local adaptation likely happened despite strong gene flow within clusters. However, this pattern can be confirmed only for the temperature gradient, because the cline was insignificant for precipitation (Figure 2b). The 18 consensus outlier SNPs that were either associated with temperature or precipitation were capable of discriminating between two clusters of provenances, which could be clearly assigned to one coastal and one inland group (Figure 7). This underpins that these SNPs have most likely featured phenotypic and genetic differentiation in Douglas‐fir beyond neutral processes such as isolation by distance. Interestingly, provenances D'Arcy (BC) and Snowqualmie Pass (WA) appeared not any longer as single subpopulation clusters when the outlier dataset was used for clustering (compare with Figure 1a with the neutral dataset of 1,500 SNPs). One possible explanation could be that isolation of these provenances occurred relatively late compared with climatic adaptation. This finding would be corroborated by the fact that both populations showed a rather expected phenotypic signal, which was similar as for the other coastal provenances (Figure 2). Coastal and interior Douglas‐fir varieties have most likely diverged during orogeny of the Cascade range, which have led to xerification of the Great Basin during the Pliocene around 2 Ma ago (Brunsfeld et al., 2001; Gugger et al., 2010). However, population contraction and expansion around the last LGM 18 ka ago could have caused local spots with limited gene exchange, in particular in areas with high mountain barriers (Gugger & Sugita, 2010).

Nevertheless and despite the overall small number of adaptive SNPs that was found, we were able to extract two “hot candidate SNPs” associated with temperature regime at seed origin. Minor allele frequencies of both SNPs showed significant correlation with mean warmest month temperature in space, which is highly correlated with the initial response climate variable from the common garden (correlation of July temperature and MWMT = 0.99). In both cases, the frequency of the minor C allele increased toward inland areas with lower temperature. In light of the strict filtering applied in this study and given the number of employed algorithms, the evidence strongly suggests that these SNPs could have featured adaptation to temperature regimes in Douglas‐fir across the steep gradient that was analyzed here. Although the correlation between allele frequencies and temperature regime was moderate and characterized by a few unexpected outliers, it is likely that the rather small sample size of analyzed trees within provenances can be responsible for that. We hence see this result as a starting point for further investigations including also landscape genomics in order to corroborate these findings with more data.

### 4.3 Isolation by environment (IBE) versus isolation by distance (IBD) and isolation by colonization (IBC)

We used redundancy analysis in order to disentangle the various drivers of among‐population differentiation by including climate, geography, and ancestry as predictors. While this approach has been shown to be very informative in two other conifers for identifying sources of differentiation (Nadeau et al., 2016), it yielded only very little insights in our study. Surprisingly, neither environment nor geography nor ancestry explained literally any variation when applied to the entire set of SNPs. Nadeau et al. (2016) argued that the high proportion of unexplained variation in *Pinus strobus* and *P. monticola* could have been caused by environmental drivers that are usually too complex to be taken into account in such studies (e.g., soil or biotic environment). In addition, the environmental gradient and number of populations sampled across that gradient are probably still too narrow in our study to highlight such contrasts. Nevertheless, when applied to the subset of 18 outlier SNPs, climate PC1 that relates strongly to the temperature regime at seed origin was at least moderately significant at *α* < 0.1, and therefore, we presume that testing these SNPs in a larger number of populations in landscape genomic studies could shed more light into adaptive population differentiation in Douglas‐fir.

### 4.4 Putative biological functions of candidate SNPs

In total, 11 out of 18 SNPs were directly located either within known genes or in close proximity to those genes, which underline their biological importance. Although not all of these genes refer directly to biological functions related to climate, their further interrogation could be nevertheless promising, since some of the found transcription factors and proteins are involved in carbohydrate metabolism and signaling pathways. For example, TIFY proteins (SNP#13432) are involved in regulation of jasmonic acid signaling pathways in *Arabidopsis thaliana* (Chung & Howe, 2009). Jasmonic acid, in turn, is an important driver of plant response to abiotic stress (e.g., Ruan et al., 2019). Bifunctional UDP‐glucose 4‐epimerase and UDP‐xylose 4‐epimerase 1 are important catalytic proteins in the pathway of cell wall polysaccharide biosynthesis and cell wall organization in stem tissue of higher plants (Rösti et al., 2007), which is an important trait in conifers conferring drought resistance (Isaac‐Renton et al., 2018).

Most interestingly, the triple‐found SNP #78509 is in very close proximity (<200 bp) to Douglas‐fir gene PSME_16548, which codes for a WD repeat‐containing LWD1 protein. This protein belongs to the circadian clock protein family and is regulating circadian period length and photoperiodic flowering in *Arabidopsis spec*. (Wang et al., 2011; Wu et al., 2008). A closely related protein of this family with similar gene ontology (Appendix S3), named GIGANTEA‐5, is involved in adaptation to temperature regimes (Cao et al., 2005) and was found to be under strong selection in *Populus balsamifera* (Fitzpatrick & Keller, 2015; Keller et al., 2012). In the study by Fitzpatrick and Keller (2015), turnover in frequency of SNPs located in this gene was best explained by differences in temperature regimes among provenance origins, which strongly corroborates the findings of this study. Although the candidate SNP may not directly alter the protein itself, it seems plausible that it influences its expression given its close adjacency. We see this finding hence as a putative needle in the haystack, which could be the starting point for further investigations at landscape level.

### 4.5 Douglas‐fir genomic resources for adaptive forest management

Douglas‐fir is currently discussed as a promising substitute species outside its natural range given its putatively higher drought tolerance and yield (Chakraborty et al., 2016; Eilmann & Rigling, 2012). Our results could be pertinent for future studies and applications, which are aiming at identifying adapted seed sources for Douglas‐fir. For instance, SNP information can be used in order to genotype large arrays of trees from progeny trials or provenance tests in order to decide whether the selected material is generally site‐adapted or likely to be maladapted to temperature and drought regime. Additionally, our dataset can be used as reference dataset for genomic assignment of individuals or stands given that many Douglas‐fir seed stands in Europe are still without confirmed geographic origin. Therefore, we provide detailed SNP and phenotypic information such as genomic positions, flanking sequences, and rank scores for more than 17,000 SNPs obtained from the various programs in Appendix S4.

## 5 CONCLUSIONS

Based on a steep phenotypic cline observed in a common garden experiment, we were able to disentangle adaptive signals in Douglas‐fir from those that were simply caused by neutral demographic processes. We showed that combining dendroclimatological data with genomic information can lead to valuable insights into the adaptation history of a widespread conifer. Although a very small fraction of the analyzed polymorphisms stood out as adaptive candidates, their functional interrogation strongly suggests that SNPs could have indeed featured climatic adaptation in Douglas‐fir. Therefore, we shed new light into the adaptive history of another conifer with high economic and ecological importance.

## CONFLICT OF INTEREST

The authors declare no conflict of interest.

## AUTHOR CONTRIBUTION

**Jan‐Peter George:** Data curation (lead); Formal analysis (lead); Investigation (lead). **Silvio Schueler:** Conceptualization (equal). **Michael Grabner:** Conceptualization (equal); Funding acquisition (equal). **Sandra Karanitsch‐Ackerl:** Data curation (equal). **Konrad Mayer:** Data curation (equal). **Michael Stierschneider:** Data curation (equal). **Lambert Weissenbacher:** Data curation (equal). **Marcela van Loo:** Conceptualization (lead); Funding acquisition (lead); Project administration (lead).

## Supporting information

Figure S1

Figure S2

Appendix S1

Appendix S2

Appendix S3

Appendix S4

## Data Availability

The raw data and additional material will be available under the Zenodo.org Digital Repository under http://doi.org/10.5281/zenodo.4714326.
