# Supplementary material for: Looking for the needle in a downsized haystack: Whole‐exome sequencing unravels genomic signals of climatic adaptation in Douglas‐fir (Pseudotsuga menziesii)
Source: Ecol Evol. 2021 May 17;11(12):8238–53. doi: 10.1002/ece3.7654 (PMC8216971; doi:10.1002/ece3.7654)
Supplement: Supplementary file 1 — Figure S1 [file ECE3-11-8238-s004.docx]

**Figure S1:** Method for discriminating early-from latewood boundaries
