# Supplementary figures and images for: Looking for the needle in a downsized haystack: Whole‐exome sequencing unravels genomic signals of climatic adaptation in Douglas‐fir (Pseudotsuga menziesii)
Source: Ecol Evol. 2021 May 17;11(12):8238–53. doi: 10.1002/ece3.7654 (PMC8216971; doi:10.1002/ece3.7654)

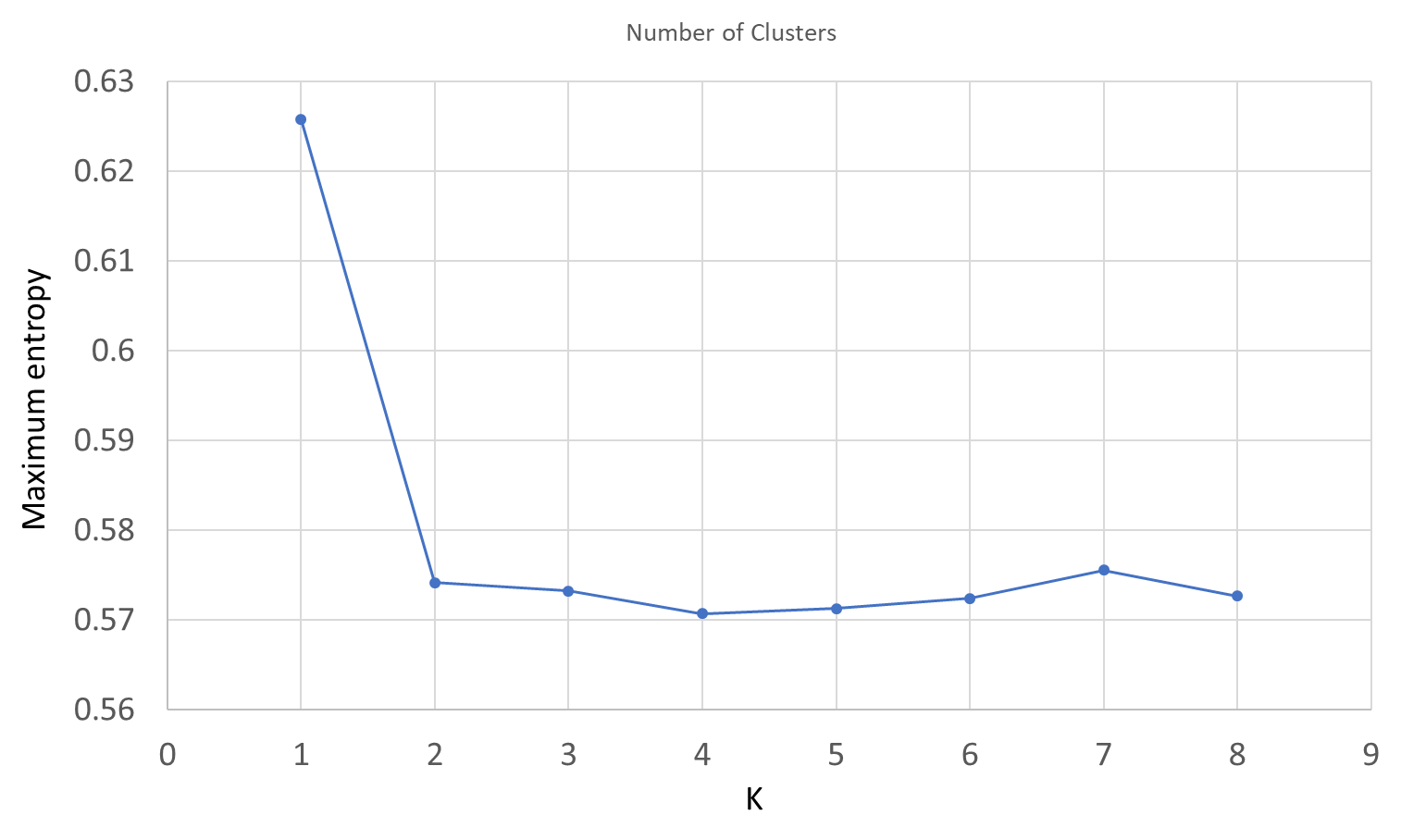

Supplement: Supplementary file 2 — Figure S2 [file ECE3-11-8238-s005.docx]

## Slide 1
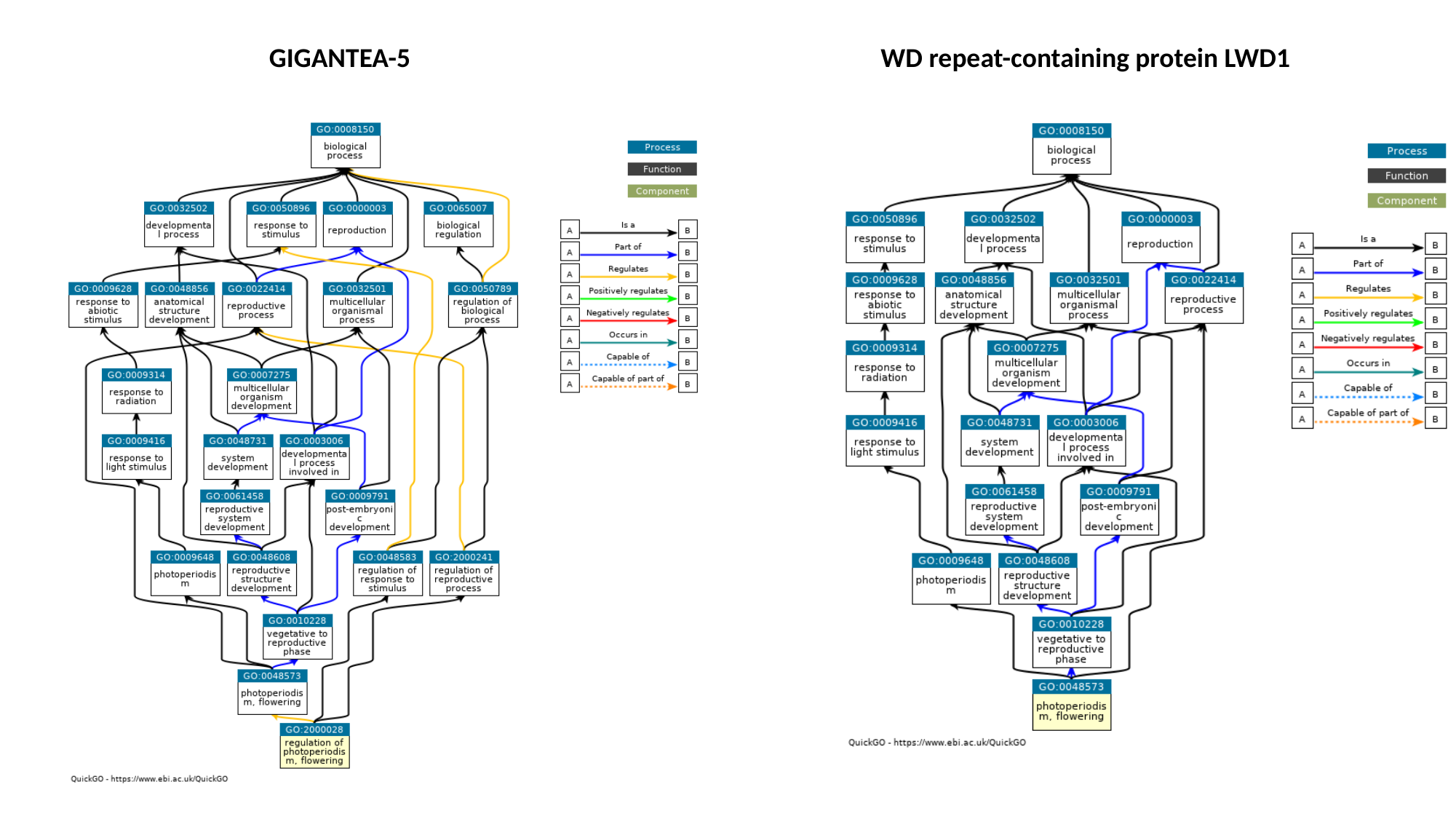

GIGANTEA-5
WD repeat-containing protein LWD1

## Slide 2
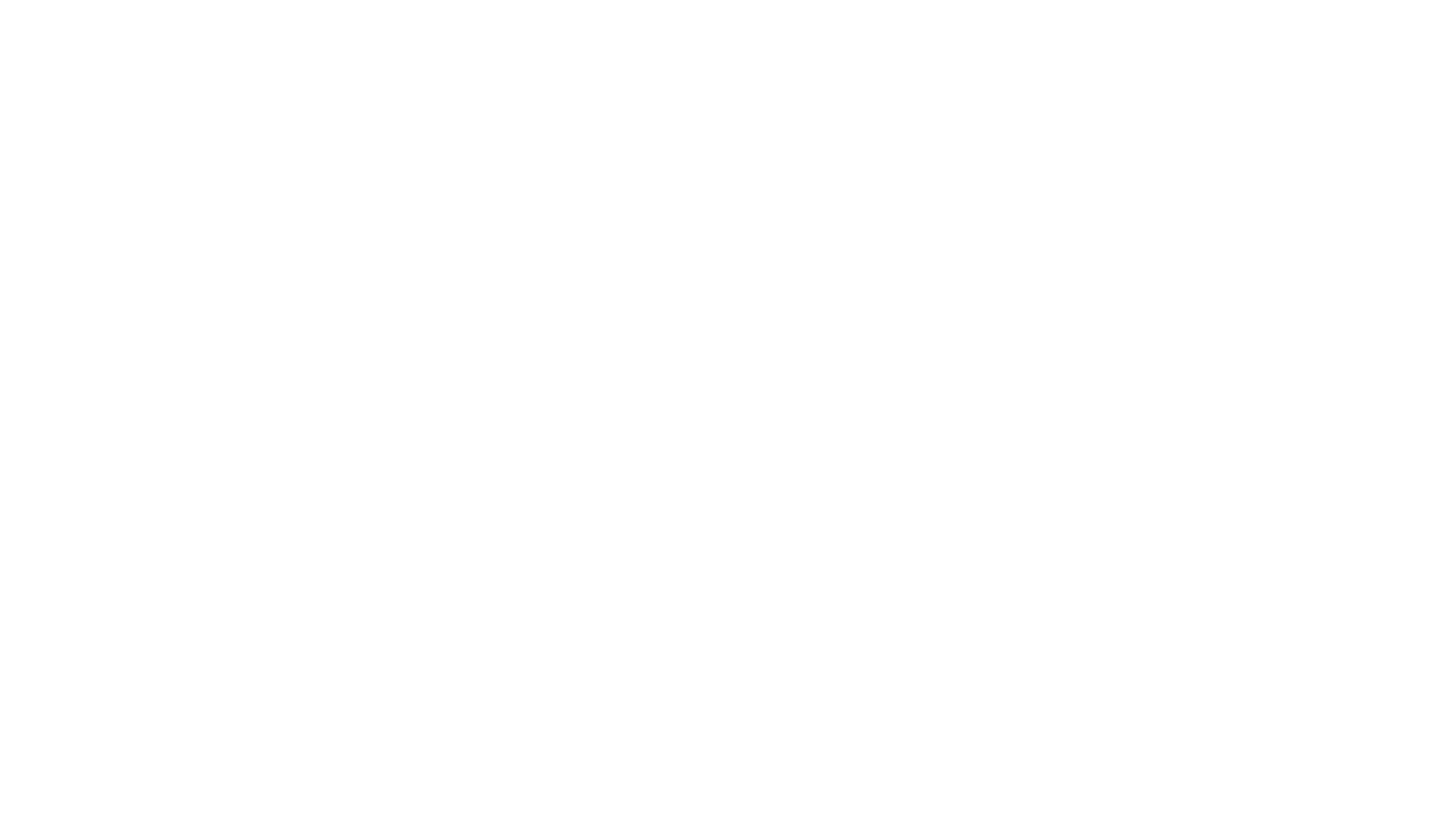

Supplement: Supplementary file 5 — Appendix S3 [file ECE3-11-8238-s002.pptx]
