# Supplementary material for: Looking for the needle in a downsized haystack: Whole‐exome sequencing unravels genomic signals of climatic adaptation in Douglas‐fir (Pseudotsuga menziesii)
Source: Ecol Evol. 2021 May 17;11(12):8238–53. doi: 10.1002/ece3.7654 (PMC8216971; doi:10.1002/ece3.7654)
Supplement: Supplementary file 3 — Appendix S1 [file ECE3-11-8238-s003.docx]

**Climate variables**

**1) Annual variables:**

*Directly calculated annual variables:*

MAT mean annual temperature (°C),

MWMT mean warmest month temperature (°C),

MCMT mean coldest month temperature (°C),

TD temperature difference between MWMT and MCMT, or continentality (°C),

MAP mean annual precipitation (mm),

MSP May to September precipitation (mm),

AHM annual heat-moisture index (MAT+10)/(MAP/1000))

SHM summer heat-moisture index ((MWMT)/(MSP/1000))

*Derived annual variables:*

DD<0 degree-days below 0°C, chilling degree-days

DD>5 degree-days above 5°C, growing degree-days

DD<18 degree-days below 18°C, heating degree-days

DD>18 degree-days above 18°C, cooling degree-days

NFFD the number of frost-free days

FFP frost-free period

bFFP the day of the year on which FFP begins

eFFP the day of the year on which FFP ends

PAS precipitation as snow (mm) between August in previous year and July in current year

EMT extreme minimum temperature over 30 years

EXT extreme maximum temperature over 30 years

Eref Hargreaves reference evaporation (mm)

CMD Hargreaves climatic moisture deficit (mm)

MAR mean annual solar radiation (MJ m^‐2^ d^‐1^)

RH mean annual relative humidity (%)

**2) Seasonal variables:**

*Seasons:*

Winter (_wt): Dec. (prev. yr) - Feb for annual, Jan, Feb, Dec for normals

Spring (_sp): March, April and May

Summer (_sm): June, July and August

Autumn (_at): September, October and November

*Directly calculated seasonal variables:*

Tave_wt winter mean temperature (°C)

Tave_sp spring mean temperature (°C)

Tave_sm summer mean temperature (°C)

Tave_at autumn mean temperature (°C)

Tmax_wt winter mean maximum temperature (°C)

Tmax_sp spring mean maximum temperature (°C)

Tmax_sm summer mean maximum temperature (°C)

Tmax_at autumn mean maximum temperature (°C)

Tmin_wt winter mean minimum temperature (°C)

Tmin_sp spring mean minimum temperature (°C)

Tmin_sm summer mean minimum temperature (°C)

Tmin_at autumn mean minimum temperature (°C)

PPT_wt winter precipitation (mm)

PPT_sp spring precipitation (mm)

PPT_sm summer precipitation (mm)

PPT_at autumn precipitation (mm)

RAD_wt winter solar radiation (MJ m^‐2^ d^‐1^)

RAD_sp spring solar radiation (MJ m^‐2^ d^‐1^)

RAD_sm summer solar radiation (MJ m^‐2^ d^‐1^)

RAD_at autumn solar radiation (MJ m^‐2^ d^‐1^)

*Derived seasonal variables:*

DD_0_wt winter degree-days below 0°C

DD_0_sp spring degree-days below 0°C

DD_0_sm summer degree-days below 0°C

DD_0_at autumn degree-days below 0°C

DD5_wt winter degree-days below 5°C

DD5_sp spring degree-days above 5°C

DD5_sm summer degree-days above 5°C

DD5_at autumn degree-days above 5°C

DD_18_wt winter degree-days below 18°C

DD_18_sp spring degree-days below 18°C

DD_18_sm summer degree-days below 18°C

DD_18_at autumn degree-days below 18°C

DD18_wt winter degree-days below 18°C

DD18_sp spring degree-days above 18°C

DD18_sm summer degree-days above 18°C

DD18_at autumn degree-days above 18°C

NFFD_wt winter number of frost-free days

NFFD_sp spring number of frost-free days

NFFD_sm summer number of frost-free days

NFFD_at autumn number of frost-free days

PAS_wt winter precipitation as snow (mm)

PAS_sp spring precipitation as snow (mm)

PAS_sm summer precipitation as snow (mm)

PAS_at autumn precipitation as snow (mm)

Eref_wt winter Hargreaves reference evaporation (mm)

Eref_sp spring Hargreaves reference evaporation (mm)

Eref_sm summer Hargreaves reference evaporation (mm)

Eref_at autumn Hargreaves reference evaporation (mm)

CMD_wt winter Hargreaves climatic moisture deficit (mm)

CMD_sp spring Hargreaves climatic moisture deficit (mm)

CMD_sm summer Hargreaves climatic moisture deficit (mm)

CMD_at autumn Hargreaves climatic moisture deficit (mm)

RH_wt winter relative humidity (%)

RH_sp winter relative humidity (%)

RH_sm winter relative humidity (%)

RH_at winter relative humidity (%)

**3) Monthly variables**

*Primary monthly variables:*

Tave01 – Tave12 January - December mean temperatures (°C)

TMX01 – TMX12 January - December maximum mean temperatures (°C)

TMN01 – TMN12 January - December minimum mean temperatures (°C)

PPT01 – PPT12 January - December precipitation (mm)

RAD01 – RAD12 January - December solar radiation (MJ m^‐2^ d^‐1^)
